# Discovery of the Elusive Leptin in Birds: Identification of Several ‘Missing Links’ in the Evolution of Leptin and Its Receptor

**DOI:** 10.1371/journal.pone.0092751

**Published:** 2014-03-24

**Authors:** Jeremy W. Prokop, Cameron Schmidt, Donald Gasper, Robert J. Duff, Amy Milsted, Takeshi Ohkubo, Hope C. Ball, Matthew D. Shawkey, Herman L. Mays, Larry A. Cogburn, Richard L. Londraville

**Affiliations:** 1 Integrated Biosciences Program, The University of Akron, Akron, Ohio, United States of America; 2 Human and Molecular Genetics Center, Medical College of Wisconsin, Milwaukee, Wisconsin, United States of America; 3 College of Agriculture, Ibaraki University, Amimachi, Ibaraki, Japan; 4 Geier Collections and Research Center, Cincinnati Museum Center, Cincinnati, Ohio, United States of America; 5 Animal and Food Sciences, University of Delaware, Newark, Delaware, United States of America; Laboratoire de Biologie du Développement de Villefranche-sur-Mer, France

## Abstract

Leptin is a pleiotropic protein best known for regulation of appetite and fat storage in mammals. While many leptin orthologs have been identified among vertebrates, an authentic leptin in birds has remained elusive and controversial. Here we identify leptin sequence from the Peregrine falcon, *Falco peregrinus* (pfleptin), and identify sequences from two other birds (mallard and zebra finch), and ‘missing’ vertebrates (elephant shark, alligator, Indian python, Chinese soft-shelled turtle, and coelacanth). The pattern of genes surrounding *leptin* (*snd1*, *rbm28*) is syntenic between the falcon and mammalian genomes. Phylogenetic analysis of all known leptin protein sequences improves our understanding of leptin’s evolution. Structural modeling of leptin orthologs highlights a highly conserved hydrophobic core in the four-helix cytokine packing domain. A docked model of leptin with the leptin receptor for Peregrine falcon reveals several conserved amino acids important for the interaction and possible coevolution of leptin with its receptor. We also show for the first time, an authentic avian leptin sequence that activates the JAK-STAT signaling pathway. These newly identified sequences, structures, and tools for avian leptin and its receptor will allow elucidation of the function of these proteins in feral and domestic birds.

## Introduction

Leptin is a well-characterized protein hormone best known for its role in appetite control and lipid metabolism [1]–[3]. In the face of epidemic obesity, several groups have investigated leptin function among vertebrates [4]–[7]. In contrast with mammals, leptin in non-mammals may not affect food intake [8], and may [9] or may not [10] be expressed from adipose tissue. The dramatic effects of leptin on fat metabolism might be a synapomorphic trait not shared by all vertebrates, while other functions (i.e., effects on immune and reproductive systems [11], [12]) are dominant in lower vertebrates. Addressing this hypothesis has been hampered by the lack of verified leptin homologues in several vertebrate taxa [4]. Arguably, the largest impediment to our understanding of leptin evolution is in the Class Aves. Early unvalidated candidates for avian leptin (Table 1) have an unrealistic sequence identity to murine leptin, rather than more closely related species, such as lizards and amphibians [13], [14]. More importantly, the *leptin* gene has not been mapped to the assembled genome sequence of any bird, especially domestic fowl [13]–[17]. Docking simulations of leptin receptor with putative avian leptin sequences do not form stabile complexes and they lack conserved leptin-leptin receptor coevolution of interacting residues [18]. We used the known protein structures for leptin [19] and leptin receptor [20], [21] to model ligand-receptor interactions and more reliably predict the true avian leptin sequence. In contrast with the lack of bird leptin sequence, bird genomes contain a highly conserved leptin receptor [22]–[24] capable of activating the JAK-STAT pathway [25], and the hypothalamic *leptin receptor* gene is responsive to divergent genetic selection for extremes of abdominal fatness [26].

**Table 1:** Sequences of *leptin* from birds.

Artifactual avian Leptin sequences	*Gallus gallus* (AF012727 & AF432509 ), *Anas platyrhynchos* (AY555727), *Meleagris gallopavo* (AF082501)
RNA sequences for avian Leptin	*Taeniopygia guttata* (XM_004175791 ), *Anas platyrhynchos* (SRR797835.67134665.2)
DNA sequences for avian Leptin	*Falco peregrinus* (AKMT01018336 & AKMT01018335), *Falco cherrug* (AKMU01055767 & AKMU01055766), *Taeniopygia guttata* (NW_002233811 & ABQF01099493)

From genomic DNA, EST, and RNA sequences.

In this paper we present leptin sequences for several ‘missing nodes’ among vertebrates, including several birds, reptiles, the coelacanth, and an elasmobranch (the elephant shark). The protein sequences produce phylogenic trees that align with traditional understanding of vertebrate evolution, and exhibit a high synteny with the same genes found around *leptin* in other vertebrate genomes (including all mammals). In addition we show that leptin protein from the Peregrine falcon forms a stable complex *in silico* and *in vitro* with the leptin receptor of birds.

## Results

**Figure 1:**
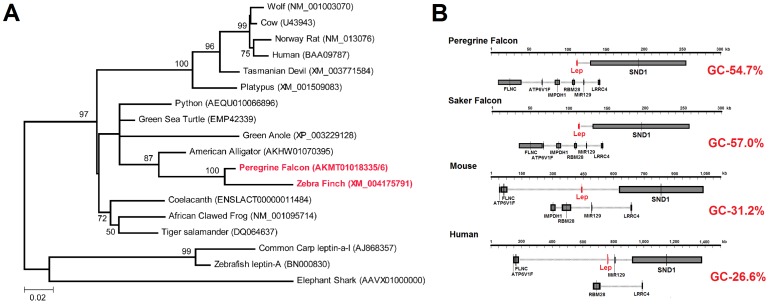
Bird Leptin gene and protein product compared to other vertebrate species. **A)** Phylogenetic analysis using Maximum Likelihood analysis of selected vertebrate species placing peregrine falcon and zebra finch leptin protein (red) most similar to alligator. **B)** *Leptin* synteny in both Peregrine and Saker falcon show chromosomal location similar to that of mouse and human. Distances for the avian species are much closer than in mouse or human, while DNA sequence for the entire region analyzed contains a higher GC content.

Sequence mining NCBI and other public genome databases identified several *leptin* DNA and RNA sequences for birds (Table 1). In addition, we identified novel *leptin* sequences for elephant shark (*Callorhinchus milii*), alligator (*Alligator mississippiensis*), Indian python (*Python molurus*), Chinese soft-shelled turtle (*Pelodiscus sinensis*), and coelacanth (*Latimeria chalumnae*) which allow, for the first time, comparison of the leptin protein from all major classes of vertebrates (Table S1, Figures S1–S2). Phylogenetic analysis of vertebrate leptin places these new sequences in relationships consistent with our current understanding of vertebrate evolution (Figure 1A). Of the avian sequences identified, only leptin from Peregrine falcon (*Falco peregrinus*) contains enough sequence information to determine the four helix cytokine structure for leptin, and was therefore chosen as the representative bird leptin sequence for further analysis. Falcon genome sequences [27] also allowed identification of high synteny of *leptin* and neighboring genes (*SND1*, *LRRC4*, *MiR129*, *RBM28*, *IMPDH1*, *ATP6V1P*, and *FLNC*) in human, mouse, and falcon species (Figure 1B).

**Figure 2:**
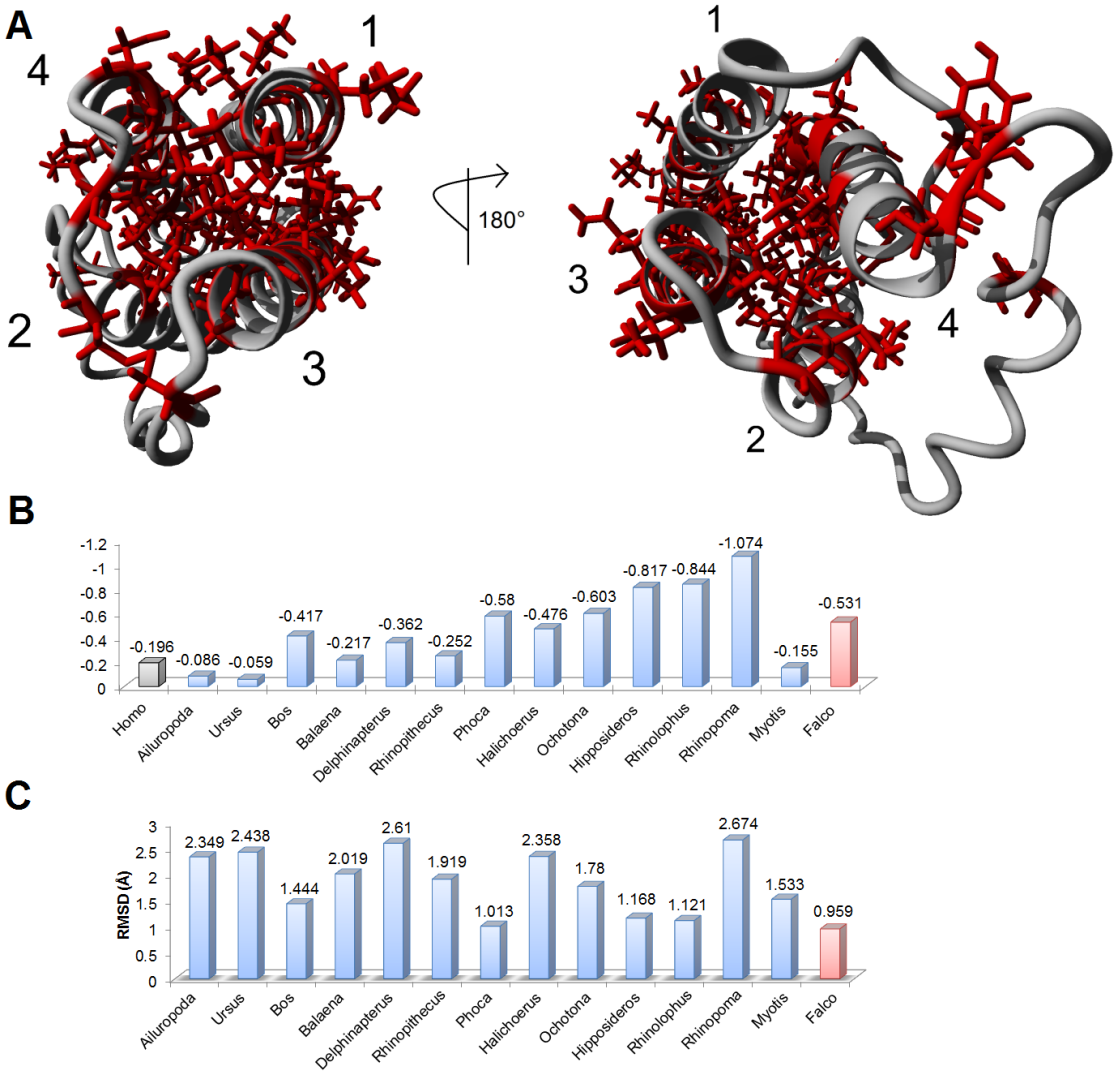
Sequences used in structurally modeling Leptin proteins throughout vertebrate evolution. **A)** Amino acids that are conserved in all 16 models shown (red) on the known structure of human leptin, with the four helices identified. The four helix bundle is conserved in all species through hydrophobic amino acids. **B)** Z-score for each model as determined using the YASARA2 force field. Values between 0 and −2 are considered to be good models. **C)** Structural alignment of each of energy minimized models to the human structure showing the average carbon alpha RMDS in angstroms (Å).

Based on tertiary structure of human leptin (pdb accession 1a × 8), sequence alignments for representative members of vertebrate leptin proteins reveal a conserved hydrophobic core of four helices with several conserved surface amino acids (Figure 2A). Homology models were created for the representative members (Figure S3). All models possessed high quality Z-scores (greater than −2.0), suggesting maintenance of fold space throughout evolution (Figure 2B). All of the models aligned to human leptin with carbon alpha root-mean squared deviations (RMSD) of 1–2.7 Å (Figure 2C).

**Figure 3:**
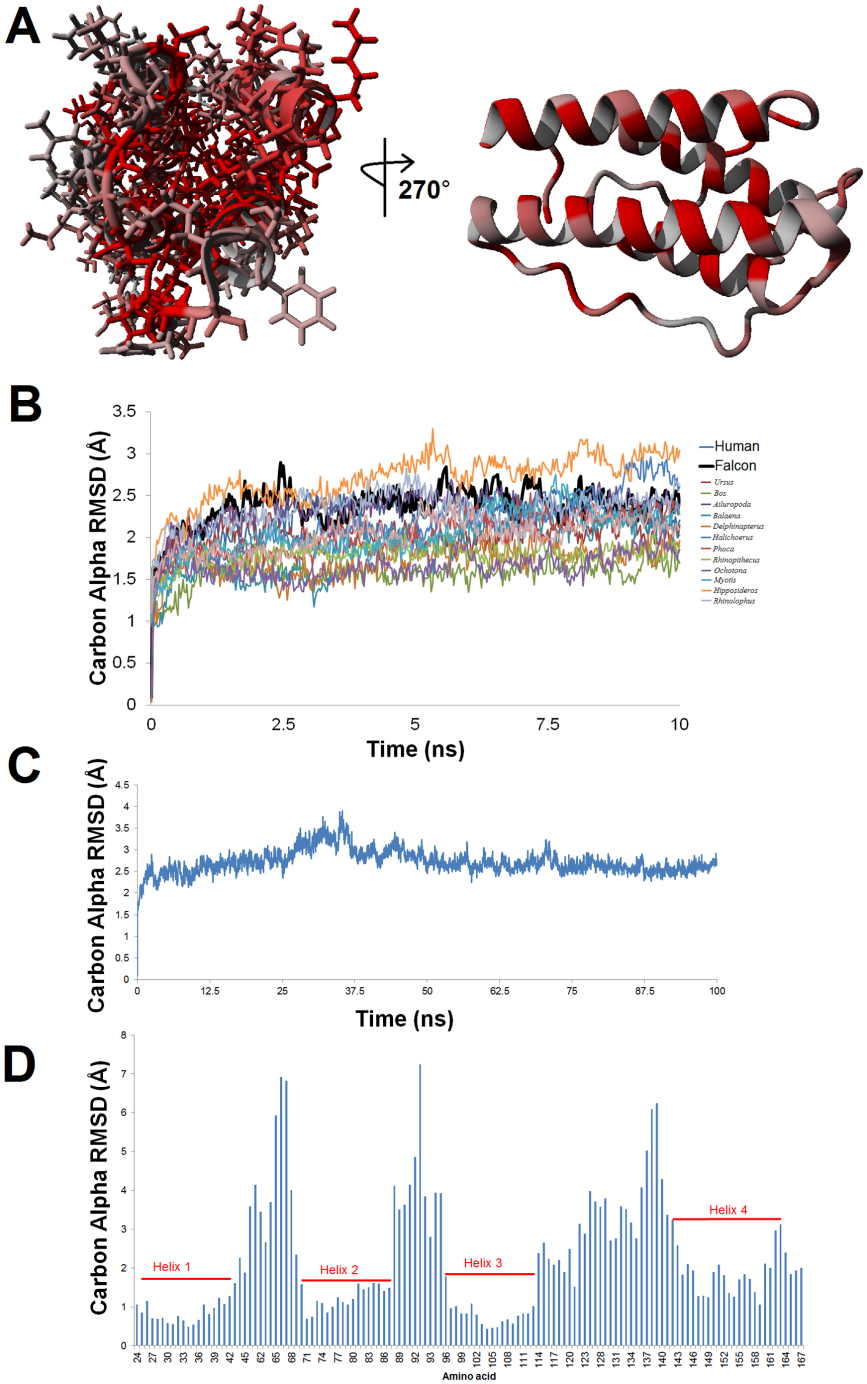
Peregrine falcon leptin model. **A)** Structural model of Peregrine falcon leptin showing conserved (red) or variant (gray) amino acids between human and peregrine falcon. **B)** Molecular dynamic simulations for 10 nanoseconds (ns) of various leptin models including the falcon (black). Plots are shown as the average movement of the carbon alpha backbone at each step in time. **C)** The carbon alpha RMSD showing the molecular movement to stabilize in the first few nanoseconds and stay around 3Å over the entire simulation. **D)** Average movement of each amino acid over the entire simulation shows stability of the four helices with higher dynamics in the loops.

The model for Peregrine falcon leptin (Figure 3A) is consistent with other vertebrate leptin proteins in molecular dynamic simulations (Figure 3B). Both the energy profile (Figure S4) and RMSD (Figure 2C) show stability of the four helix cytokine structure in extended molecular dynamic simulations. Analysis of the movement of individual amino acids over the entire 100 ns simulation further confirm low movement of helical packing with increased dynamics in the loops of the protein (Figure 3D). Several additional bird leptin sequences were identified (Table 1), all of which contain amino acids conserved among birds but different from other vertebrate leptin proteins. Several other partial avian/non-avian leptin sequences have been identified (Table S1), but do not contain enough sequence to determine their molecular structure relative to the known human structure. These include a short transcript of duck (*Anas platyrhynchos*) identified by mining RNA sequence reads from the recently reported duck *leptin* [28]. Translation of the RNA sequence for duck corresponds to amino acids that are conserved with the Peregrine falcon leptin sequence (Figure S5A). When mapped onto the model of the Peregrine falcon leptin these amino acids correspond to the loop between helix 1 and helix 2. This confirms that not only is cytokine helical packing conserved between these two sequences but also loops, which are not conserved with humans and mouse. Amino acids conserved between the zebra finch and the Peregrine falcon leptin show a highly conserved hydrophobic core with many additional conserved amino acids in loops and on the surface of leptin (Figure S5B). Data mining for sequences of avian leptin has resulted in the identification of several other leptin proteins that have not yet been annotated in the genomes. These include coelacanth leptin (Figure S6–S7), python (Figure S8), alligator (Figure S9), and *Chelonia* (Figure S10).

**Figure 4:**
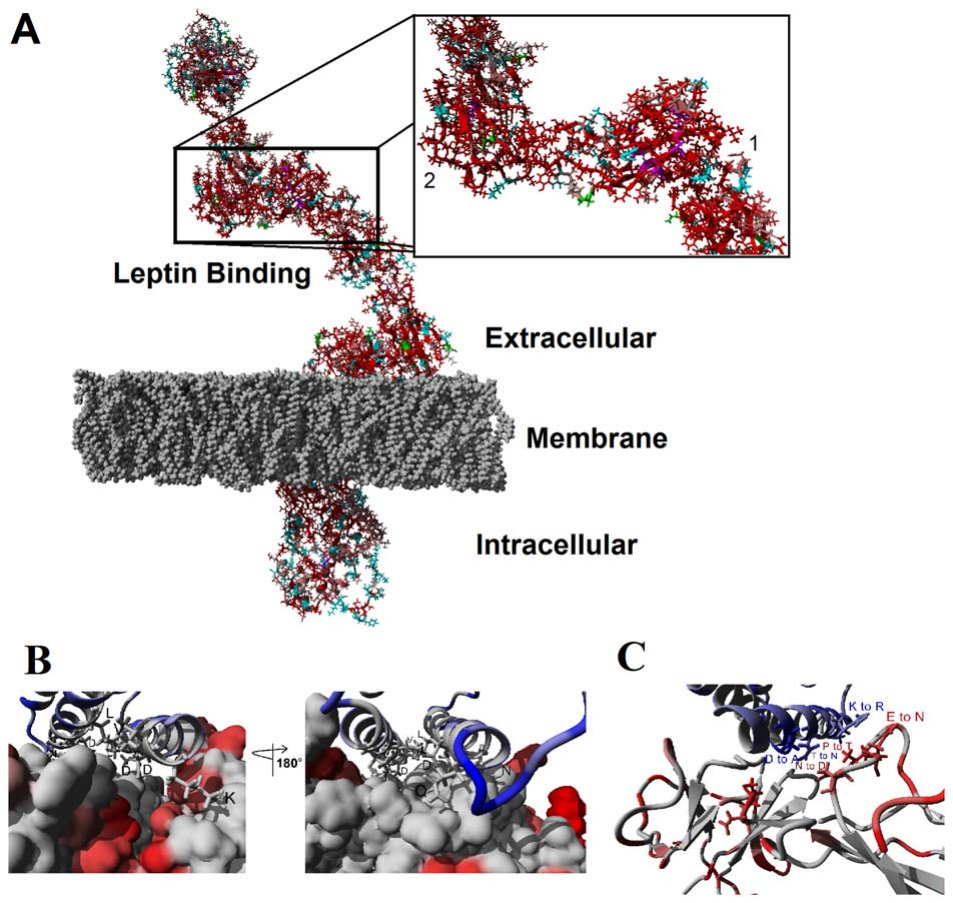
Avian leptin bound to leptin receptor. **A)** Energy minimized model of full chicken leptin receptor embedded in a lipid membrane. Amino acids colored in red are conserved amongst various avian sequences and human. Those in cyan are conserved only in avian sequences, magenta represents disulfide bonds and green those amino acids known to be N-glycosylated. Sequence alignments can be seen in the supplemental Figure S11. **B)** Energy minimized Peregrine falcon Leptin (ribbon structure) and leptin receptor (molecular surface) showing amino acids that are conserved with human in gray. Those amino acids that vary from human are shown in blue for leptin and red for leptin receptor. **C)** Complex as shown in B but with ribbon diagram for leptin receptor. View shows the coevolution between two amino acids on leptin and one on leptin receptor.

Several avian species have an annotated leptin receptor sequence. These sequences were used to identify amino acids that were conserved among birds only or with human (Figure S11). A protein model of the leptin receptor (including the extracellular domain, transmembrane domain embedded in a lipid membrane, and the intracellular domain) for chicken was created (Figure 4A). Conserved amino acids among vertebrate receptors were then mapped onto this model to identify a leptin binding site. Bird leptin receptors are comprised of unique bird-specific amino acids (Figure 4A, cyan), and those conserved between birds and humans (Figure 4A, red). Two domains highly homologous with the human leptin receptor are the second fibronectin type-III and the immunoglobin (Ig)-like domains (at the binding interface with leptin in Figure 4A). These sites each bind a separate leptin and allow for dimerization of two leptin receptor molecules [21]. The second fibronectin type-III domain contains a WSXWS motif, which is important for leptin receptor dimerization and activation [29] and is conserved among birds, humans, and fish [18]. This observation suggests conserved binding sites between leptin and its receptor across vertebrate classes. The intracellular (N-terminal) domain of bird leptin is less homologous to human and other mammals, suggesting a bird-specific JAK/STAT activation, which may contribute to the difficulty of addressing chicken STAT3 activation using mammalian cell culture [30].

Leptin receptor sequence for Peregrine falcon (Figure S12) was identified from genomic DNA and models created for receptor interaction based on previous models for the chicken receptor [18]. Ten nanoseconds of molecular dynamic simulations on either the complex (leptin-leptin receptor) or each one of the proteins alone showed both leptin and leptin receptor to have dynamics reduced (greater stability) when found in the complex (Figure S13). For both leptin and leptin receptor, many amino acids that we predict are critical to ligand-receptor interaction are conserved between falcon and human (Figure 4B). Several hydrophobic (Leu [L] and Val [V]), polar acidic (Asp [D]) and polar basic (Lys [K]) amino acids are conserved in this binding pocket. Amino acids that vary in both the leptin and receptor for peregrine reveal possible co-evolution of helix one of leptin with a loop of the receptor (Figure 4C). Two amino acids differ (Asp [D] to Ala [A] and Thr [T] to Asn [N]) between human and peregrine leptin and one (Asn [N] to Asp [D]) in leptin receptor. In human, we suggest the D of leptin to hydrogen bond with the N of the receptor while the T (small) stabilizes the bond. For falcon, the N of leptin (variation from T to N) hydrogen bonds with the D of leptin receptor (variation from N to D), while the small A (variation from D to A) stabilizes the bond.

**Figure 5:**
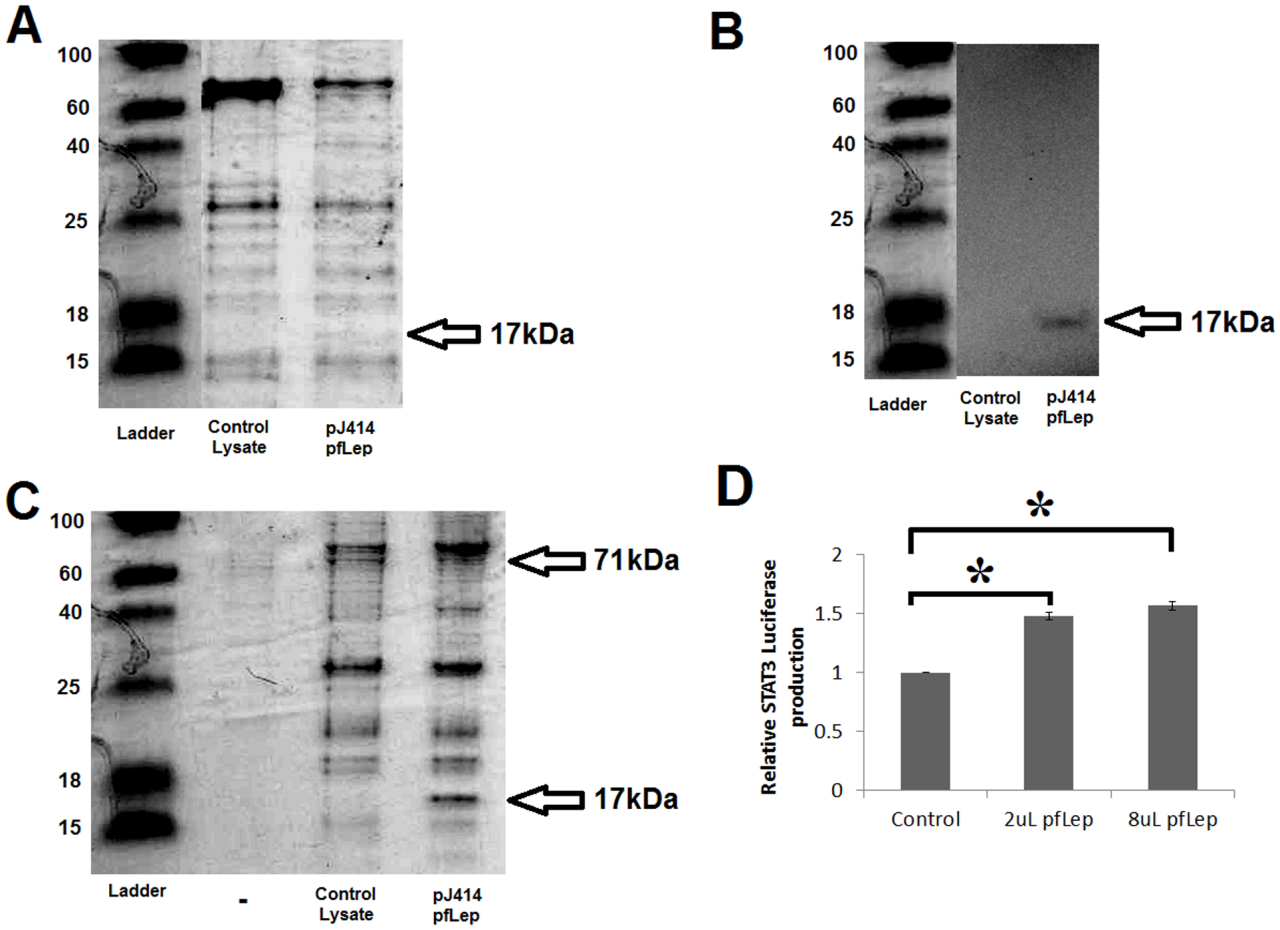
Purification of Peregrine falcon lepin (pfleptin). **A)** Coomassie stain following 15% Tris-Tricine SDS PAGE. Control lysate and lysate of the pJ414 pfleptin expressing cells were previously passed over Ni-Sepharose and dialyzed into 125 mM NaCl. 15 μL of each sample were loaded onto the gel. The pfleptin protein with tag is 17 kDa. **B)** Western blot of A following transfer to membrane and probed with an anti-His primary antibody. **C)** Glutathione pull down of the pGEX4T-chLepR(227–628) which can be seen at 71 kDa. Beads were then incubated with either the control lysate or the pfleptin lysate. This resulted in concentrating the pfleptin protein at 17 kDa. **D)** Luciferase assays showing activation of the STAT pathway of chicken leptin receptor transfected CHO cells treated with concentrated peregrine falcon leptin (n  =  2). Error bars represent the standard error of the mean and * represents a p-value ≤ 0.005 between samples. No significance was seen between the two concentrations of leptin treatment suggesting maximum saturation in the assay was reached.

To determine if Peregrine falcon leptin can be expressed in a soluble form, we had the sequence codon optimized and cloned into multiple bacterial expression vectors (Figure S14). Following induction, lysis and a His-trap column of the pJ414-6 × His-TEV-pfLep(23–164) construct, a protein of the expected size (17 kDa) was produced (Figure 5A). The protein was further identified in a western blot using an anti-His probe (Figure 5B). This 17 kDa protein was further concentrated when pulled down using a GST-tagged chicken leptin receptor protein (Figure 5C). This Peregrine falcon leptin protein activates the chicken leptin receptor *in vitro* as determined through STAT3 driven luciferase production in CHO cells (Figure 5D).

## Discussion

We assert that the falcon leptin sequences represent the first true bird leptin sequences identified. These sequences align with other vertebrate leptin proteins in a way consistent with vertebrate phylogeny, and are consistent with the pattern of gene arrangement for all known *leptin* genes. Falcon leptin also forms stable complexes with established bird leptin receptors both *in-silico* and *in-vitro*. A duck *leptin* transcript was recently reported from lung tissue after immune system activation [28]. The protein coded by this RNA sequence demonstrates high conservation to our annotated Peregrine falcon leptin in regions outside the four cytokine helices, suggesting it is not a related cytokine but is in fact a *leptin* transcript. Several factors may have contributed to the previous inability to identify bird leptin. We suggest that the expression pattern among birds is distinct from mammals; as is the case in fish [11] and reptiles [31], suggesting that many researchers have looked in the wrong tissues for identification of transcripts. In addition, the genomic sequences of Peregrine and Saker falcons contain an exceptionally high GC content in and around the *leptin* gene (Figure 1B), and thus represent a high probability of epigenetic regulation. This would correspond with tightly bound histones in avian tissues not expressing leptin, which would likely complicate the purification of DNA and genomic sequencing of leptin. Use of these new avian leptin sequences will allow examination in multiple bird species. The tools and vectors designed in this paper will serve as a helpful beginning to further characterize leptin in birds. Despite numerous large sequencing projects accruing more than 600 K expressed sequence tag (EST) sequences and repeated assembly of the chicken genome sequence, *leptin* gene is missing from the chicken genome and that of two other domestic birds (duck and turkey). More than 100 papers have been published on leptin in the chicken or some other bird species based on measurements of *leptin* expression as a gene, protein or a variety of biological responses to exogenous human or murine leptin. Our description of the structure and evolution of the first *bona fide* avian *leptin* gene and its encoded leptin protein represents a critical first step toward understanding the distribution and function of leptin among birds. We suggest and provide the tools for further *in vitro* and *in vivo* validations of the bird leptin, such as additional luciferase assays using the newly designed bird specific STAT3 assay [30]. In this paper we have performed a genome analysis followed by gene sequence identification and protein structure to protein function analysis (genome-to-sequence-to-structure-to-function, or G-S-S-F for short) to validate a potential gene of interest to a research community. Approaches such as this can be applied to many other gene discoveries or validation to understand the context in which genomes and proteins have evolved.

## Methods

### Bioinformatics and sequence analysis

Protein sequences for leptin were identified from translated DNA sequences for *Latimeria chalumnae* (coelacanth, ENSLACT00000011484), and *Chelonia mydas* (green sea turtle, EMP42339). These sequences were then queried against the *Alligator mississippiensis* (American Alligator) raw genome data obtained from the crocodilian genome project [32]. All BLAST [33] analysis was performed using the BLASTALL standalone suite, Version 2.2.26 with default arguments. Alignments were performed using the Probcons [34] standalone Version 1.12 with default arguments. The consensus sequence was blasted against the *Falco peregrinus* (Peregrine falcon) and *Falco cherrug* (Saker falcon) whole genome FASTA files [27]. Roughly 150–300 kB of sequence flanking both directions of the identified *leptin* gene in both *Falco peregrinus* and *Falco cherrug* was then obtained from the genomic assembly. All ambiguous base calls from the genomic assembly were masked. The Falcon sequences were queried against the nucleotide database, resulting in identification of the *Taeniopygia guttata* partial leptin sequences (zebra finch, NW_002233811.1 and ABQF01099493.1). The sequence of exon II partial transcript from *Taeniopygia guttata* (XM_004175791) was identified by nucleotide BLAST against the refseq_RNA database. *Anas platyrhynchos* sequence reads [28] were queried to identify the most homologous read to *Falco peregrinus* leptin sequence resulting in the identification of read SRR797835.67134665.2 from the SRA071775 dataset. Leptin receptor sequences for multiple avian species were obtained by BLAST against the nucleotide database using the chicken mRNA sequence (NM_204323). DNA sequences were manually spliced and translated.

Genes assembled within leptin-containing scaffolds of Peregrine falcon and Saker falcon were identified by querying each scaffold against the nucleotide (nt) database. Expected value cutoff was e0.0, and high-scoring segment-pair return cutoff was set to 1000, with all other arguments default. The following gene set was identified, shown here with the human accession numbers obtained from the nt database: *SND1* (NM_014390.2), *LRRC4* (NM_022143.4), *MiR129* (NR_029596.1), *RBM28* (NM_018077.2), *IMPDH1* (NM_001142576.1), *ATP6V1P* (CR456896.1), and *FLNC* (NM_001458.4). Gene synteny was analyzed by copying scaffolds from the selected genomes that had positive hits for human *MiR129* into a local database. Each of the aforementioned sequences was then blasted using both blastn and tblastn sub-programs for regional consensus, and the terminal regions were exported. GC content for each scaffold was calculated using gccalc perl script obtained from CPAN (www.cpan.org).

### Evolutionary analysis

Evolutionary analyses were conducted in MEGA5 [35]. Initial multiple sequence alignments of representative vertebrate leptin amino acid sequences were performed in MEGA5 using ClustalW. This was followed by manual alignment informed by protein structural homologies, i.e. use of the predicted location of the four helices and hydrophobic amino acids that contribute to the core packing of the protein. Models resulting in the lowest Bayesian Information Criterion (BIC) score for the JTT+G model of evolution [36] were used. This model was then employed in Maximum Likelihood analyses on two data sets (18 and 26 taxa). 1000 bootstrap repetitions were performed on the small tree (Figure 1) while 500 were performed on the larger tree (Figure S2).

### Generation of protein models and molecular dynamics simulations

Models for multiple vertebrate leptin proteins were created using amino acid swapping of the known human leptin structure (pdb 1a × 8) leaving out the disordered loop between helix one and two. These included the *Ursus thibetanus japonicas* (Japanese black bear, AB255164), *Bos taurus* (cattle, P50595), *Ailuropoda melanoleuca* (panda, XM_002913420), *Balaena mysticetus* (bowhead whale, JN833620), *Delphinapterus leucas* (beluga whale, JN833619), *Halichoerus grypus* (grey seal, AJ618982), *Phoca vitulina* (harbor seal, AJ618981), *Rhinopithecus bieti* (black snub-nosed monkey, HM125573), *Ochotona nubrica* (Nubra pika, EF091861), *Myotis ricketti* (Rickett's big-footed Myotic, GU230846), *Hipposideros armiger* (great roundleaf bat, GU230835), *Rhinolophus ferrumiquinum* (greater horseshoe bat, GU230845), *Rhinopoma microphyllum* (Greater mouse-tailed bat, GU230830) and the *Falco peregrinus* (peregrine falcon, AKMT01018335.1). Following amino acid swapping to get the sequence for each respective species on the structure of leptin, pKa was predicted for each model with a pH of 7.4 and water inserted into a simulation square of 56 × 48 × 40Å to a density of 0.997 g/mL using YASARA (www.YASARA.org). Energy minimizations were then performed multiple times using AMBER03 force field [37] with periodic boundaries. Molecular dynamic simulations were performed on each of the models for 10 ns using the md_run macro and were analyzed with the md_analyze and md_analyzeres macros. The energy minimized structures created from the md_analyze macro for each model were structurally aligned to the human energy minimized structure using the MUSTANG algorithm [38] and the average carbon alpha RMSD calculated between the two models. Structural statistics were performed on each model by taking the energy minimized confirmation and removing the water. Then the YASARA2 force field (http://www.yasara.org/kbpotentials.htm) was used to energy minimize and the model quality determined.

A model for the full-length chicken receptor was created by independently modeling each domain of the chicken sequence (NM_204323) using the I-TASSER server [39], [40]. Models were manually joined based on the global receptor conformation as determined in cryoEM structures [21]. Following assembly of all domains the transmembrane domain was inserted into a lipid membrane using the md_runmembrane.mcr macro in YASARA. This macro inserted the complete structure into a simulation square of 140 × 318 × 157Å and created a membrane of phosphatidyl-ethanolamine (PEA) that the helical transmembrane domain transverses. This was followed by the insertion of water at 0.997 g/mL and energy minimization steps. Molecular dynamic simulations were then run for 25 ns. The energy minimized structure was used to identify the conserved amino acids between avian species and human by coloring the conserved amino acids between chicken and human alignments in red and coloring the disulfide bridges and N-glycosylation as previously reported [41]. Additionally the amino acids conserved in only the avian sequences and not the human were colored throughout the structure. A model for the Peregrine falcon leptin receptor was created for the leptin binding domain (the second fibronectin type III domain) by amino acid swapping with the chicken receptor. Leptin for Peregrine falcon was then docked to the receptor using the previously modeled complex [18]. The complex was energy minimized in water using the AMBER03 force field [37] and molecular dynamic simulations performed for 10 ns in a simulation square of 95 × 82 × 55 Å. In addition, each protein (leptin or leptin receptor) was simulated alone in the same simulation square (water at a density of 0.997 g/mL replacing the missing protein). Simulation trajectory was analyzed in the same manner as the leptin simulations.

### Cloning and expression of pfleptin and chleptin receptor

An expression vector for the Peregrine falcon leptin (pfleptin) was codon optimized and synthesized by DNA2.0 (www.dna20.com) containing BamHI and NotI restriction sites, and a 6 × His N-terminal tag that can be removed with TEV cleavage. The construct was then subcloned into pET28 and pGEX4T (GE Life Science) vectors using the restriction sites BamHI and NotI. PCR was performed using Phusion Hot Start II polymerase (Thermo Scientific) to clone the pfleptin into the pMAL-p5x (New England Biolabs). Several bacterial-inducible chicken leptin-receptor constructs were created by cloning the sequence from the pCI-chleptin receptor [25], [42] into pGEX-4T using Phusion Hot Start II polymerase and the BamHI and NotI cloning sites. All vector constructs were sequence confirmed using BigDye sequencing (Applied Biosystems). Vectors were transformed into BL21 DE3 *E. coli* (New England Biolabs). A single colony of each was inoculated into 5 mL of LB containing the appropriate antibiotic and grown overnight. This was inoculated into 500 mL of Terrific broth containing the appropriate antibiotic and grown at 37°C to an OD600 of 0.4. Cultures were induced with 0.5 mM IPTG and grown at 20°C for 12 hours. Samples were then centrifuged to collect cells, resuspended in appropriate buffers for column purification, and sonicated on ice. Following centrifugation at 16,000 x g the lysate was passed over Ni-Sepharose or glutathione Sepharose (GE Life Science) depending on the tag used for purification. Following elution of the protein, samples were concentrated and dialyzed using 5 kDa Vivaspin 2 columns (GE Life Science). Following boiling in 2x sample buffer, proteins were run on a 15% Tris-tricine SDS PAGE. Western blot for the His tag was performed by transferring the gel to a nitrocellulose membrane, blocked in 5% milk, probed with the His-probe (G-18, Santa Cruz Biotechnology), washed in PBS-T, treated with secondary donkey anti-Goat HRP antibody, washed in PBS-T, and chemiluminescence recorded on film.

Following column purification and dialysis/concentration of the GST-tagged chicken leptin receptor, fresh pull downs of the protein were performed using 40 μL of glutathione sepharose beads (GE Life Science) in 500 mL BB500 (500 mM NaCl, 20 mM Tris, 0.2 mM EDTA and 10% glycerol with total pH adjusted to 7.9). Samples were rotated and incubated at room temperature for 1 hour followed by washing the beads 5x with BB750 (750 mM NaCl, 20 mM Tris, 0.2 mM EDTA and 10% glycerol with total pH adjusted to 7.9) and 3 washes with BB500. The concentrated His-tagged pfleptin protein was then added to the beads in 500 mL BB500 and incubated at room temperature for 1 hour. Following 3 washes with BB750 and one with BB500, samples were boiled in 5x sample buffer and run on 15% Tris-tricine SDS PAGE.

### Luciferase assays

5 × 10^4^ CHO K1 (ATCC) cells were plated into each well of a 24-well plate, with Ham's-F12 supplemented with 10% fetal bovine serum (Atlanta Biologicals), 10 mmol/L HEPES, and 30 mmol/L sodium bicarbonate and cultured in a humidified 37°C incubator with 5% CO2. After 24 hrs, each well of cells was transfected with the 500 ng pCI-chLEPR, 2 μL of either positive or negative Cignal STAT3 reporter (Qiagen) constructs (containing both the luciferase vector and a control Renilla vector), 2 μL of FuGENE transfection reagent (Roche), and 38 μL of serum free Ham's-F12 media. After incubation of the complex for 10 min at room temperature, the transfection mix was added to each well one drop at a time. Cells were then incubated for 24 hrs and then washed twice with PBS. The concentrated and partially pure pfleptin protein was mixed at two concentrations into 200 μL of complete Hams-F12 media containing antibiotics. After 36 hrs in a 37°C incubator with 5% CO_2_, cells were washed twice with PBS and lysed using 100 μL per well of 1X passive lysis buffer (Promega). Luciferase assays were then performed using the Dual-Luciferase Reporter Assay System (Promega) as recommended by the manufacturer. The luminescence of each sample was read in triplicate on two independent experiments. The leptin treatment on the positive STAT3 construct (read in triplicate) was divided by the average for the same leptin treatment on the negative luciferase construct to account for any role of variation of Renilla vector or non-STAT3 dependent luciferase vector regulation by leptin treatment, yielding a highly sensitive luciferase assay. Student t-tests were performed on the two experiments with all p-values ≤ 0.005 recorded as significant.

## Supporting Information

Table S1 **Sample information for leptin and leptin receptor sequenced used in this study.**
(XLSX)

Figure S1 **Sequence alignment of 26 vertebrates that include 4 bird sequences.** These are the sequences used in the larger phylogenetic tree S2 and a subset of 18 were used for the smaller tree (Figure 1A).
(TIF)

Figure S2 **Expanded tree including all identified avian leptin sequences.** Phylogenetic analysis (Maximum Likelihood) of all known amphibian, reptile and avian leptin sequences along with other representative vertebrate leptin proteins. Numbers as nodes represent percentage of 500 bootstrap replicates. Mallard was represented by only 27 amino acids and zebra finch and Saker falcon were only represented by partial leptin sequences (see Figure S1 for alignment used for the analyses).
(TIF)

Figure S3 **Energy minimized models for each of the 16 sequences.** Models showing the amino acids that differ between human and each of the species in red.
(TIF)

Figure S4 **Extended simulation of Peregrine falcon leptin.** The energy over the entire simulation showing rapid stabilization.
(TIF)

Figure S5 **Mapping conservation of avian-specific sequence to the structure of Peregrine falcon.** **A)** Short sequence translated from the duck leptin transcript aligned to falcon. Amino acids shown correspond to the duck transcript and amino acid colors are of that in the sequence alignment. The amino acids correspond to the second helix and the segment that crosses the first helix to the second helix. **B)** Conserved amino acids (red) between the Peregrine falcon and zebra finch sequences. Sequences for the zebra finch starts at the end of helix one.
(TIF)

Figure S6 **Coelacanth leptin.** **A)** Leptin model with amino acid properties colored (yellow = hydrophobic, magenta = aromatic, red = polar acidic, blue = polar basic, green = hydrophilic) looking down the hydrophobic core. **B)** Surface plot showing the electrostatic region for interaction with receptor. **C)** Conserved amino acids (red) between coelacanth and *Chelonia* sequence shown on the structure of coelacanth. **D)** Conserved amino acids (red) between coelacanth and *Xenopus* sequence shown on the structure of coelacanth.
(TIF)

Figure S7 **Molecular dynamic simulation for Coelacanth leptin.** **A)** Energy for the Coelacanth over the 10 ns simulation. Energy is very different than the other 15 models used in analysis. **B)** Molecular dynamic simulations for coelacanth yielded carbon alpha RMSD higher than other species. Both the energy and the movement are consistent with a more open protein structure, typical of cold-bodied animals [43].
(TIF)

Figure S8 **Python leptin.** **A)** Sequence alignment of the python leptin to the Peregrine falcon, mouse and human. Amino acids conserved in python and Peregrine falcon but not the mouse and the human are highlighted in red. **B)** Amino acids identified in red in A shown on the structure of the Peregrine falcon leptin. These amino acids do not correspond to structural packing.
(TIF)

Figure S9 **Alligator leptin model.** **A)** Energy minimized model of alligator leptin showing the amino acids that are conserved with Peregrine falcon leptin shown in red. The binding site with the receptor is highly conserved between the two species. **B)** 10 ns of simulation show stability of the protein structure.
(TIF)

Figure S10 ***Chelonia*** **leptin model.** **A)** Energy minimized model of *Chelonia* leptin showing the amino acids that are conserved with Peregrine falcon leptin shown in red. The binding site with the receptor is highly conserved between the two species. **B)** 10 ns of simulation show stability of the protein structure.
(TIF)

Figure S11 **Sequence alignments of avian species with human for leptin receptor.** Amino acids colored cyan are conserved in the multiple avian sequences but not with human. Amino acids highlighted in magenta are cysteines known to form disulfide bonds. The WSXWS motif in underlined and colored red. Domains are identified by highlighting the human sequence in yellow, red or cyan. Amino acids known to be N-glycosylated are highlighted in green and those suggested to be N-glycosylated in avian sequences in light green.
(TIF)

Figure S12 **Sequences of the Peregrine and Saker falcon leptin receptor aligned to chicken.** Color coding and alignments are the same as in Figure S11.
(TIF)

Figure S13 **Simulation data for the Peregrine falcon leptin and leptin receptor.** **A)** RMSD following 10 ns simulation for leptin free (red) or complexed to the receptor (blue). **B)** RMSD following 10 ns simulation for leptin receptor free (red) or complexed to the leptin (blue). Both simulations were stabilized when found as a complex.
(TIF)

Figure S14 **Constructs used for studying avian leptin.** Restriction enzymes listed (NotI and BamHI) were used to clone each construct at positions shown on each vector. All constructs contain a cleavable tag when treated with various enzymes (Thrombin, TEV, and Factor Xa).
(TIF)
