# Supplementary figures and images for: Discovery of the Elusive Leptin in Birds: Identification of Several ‘Missing Links’ in the Evolution of Leptin and Its Receptor
Source: PLoS One. 2014 Mar 24;9(3):e92751. doi: 10.1371/journal.pone.0092751 (PMC3963946; doi:10.1371/journal.pone.0092751)

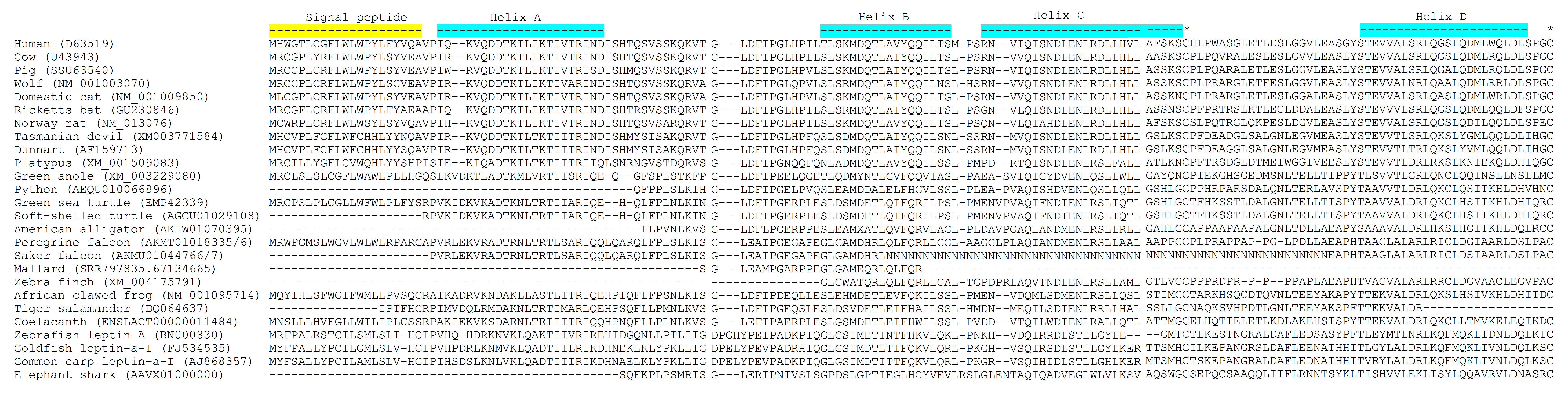

Supplement: Figure S1 — Sequence alignment of 26 vertebrates that include 4 bird sequences. These are the sequences used in the larger phylogenetic tree S2 and a subset of 18 were used for the smaller tree (Figure 1A). (TIF) [file pone.0092751.s002.tif]

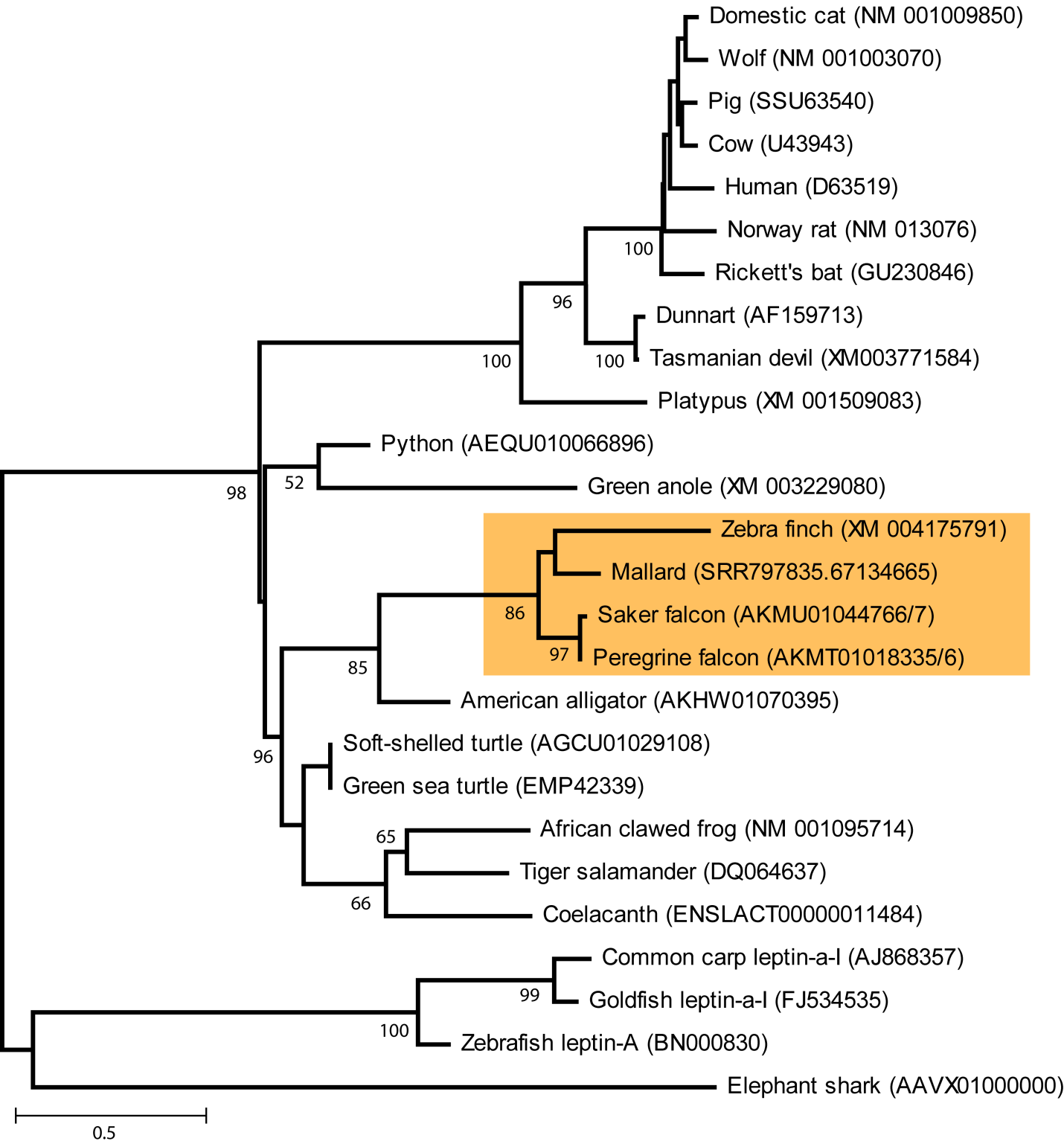

Supplement: Figure S2 — Expanded tree including all identified avian leptin sequences. Phylogenetic analysis (Maximum Likelihood) of all known amphibian, reptile and avian leptin sequences along with other representative vertebrate leptin proteins. Numbers as nodes represent percentage of 500 bootstrap replicates. Mallard was represented by only 27 amino acids and zebra finch and Saker falcon were only represented by partial leptin sequences (see Figure S1 for alignment used for the analyses). (TIF) [file pone.0092751.s003.tif]

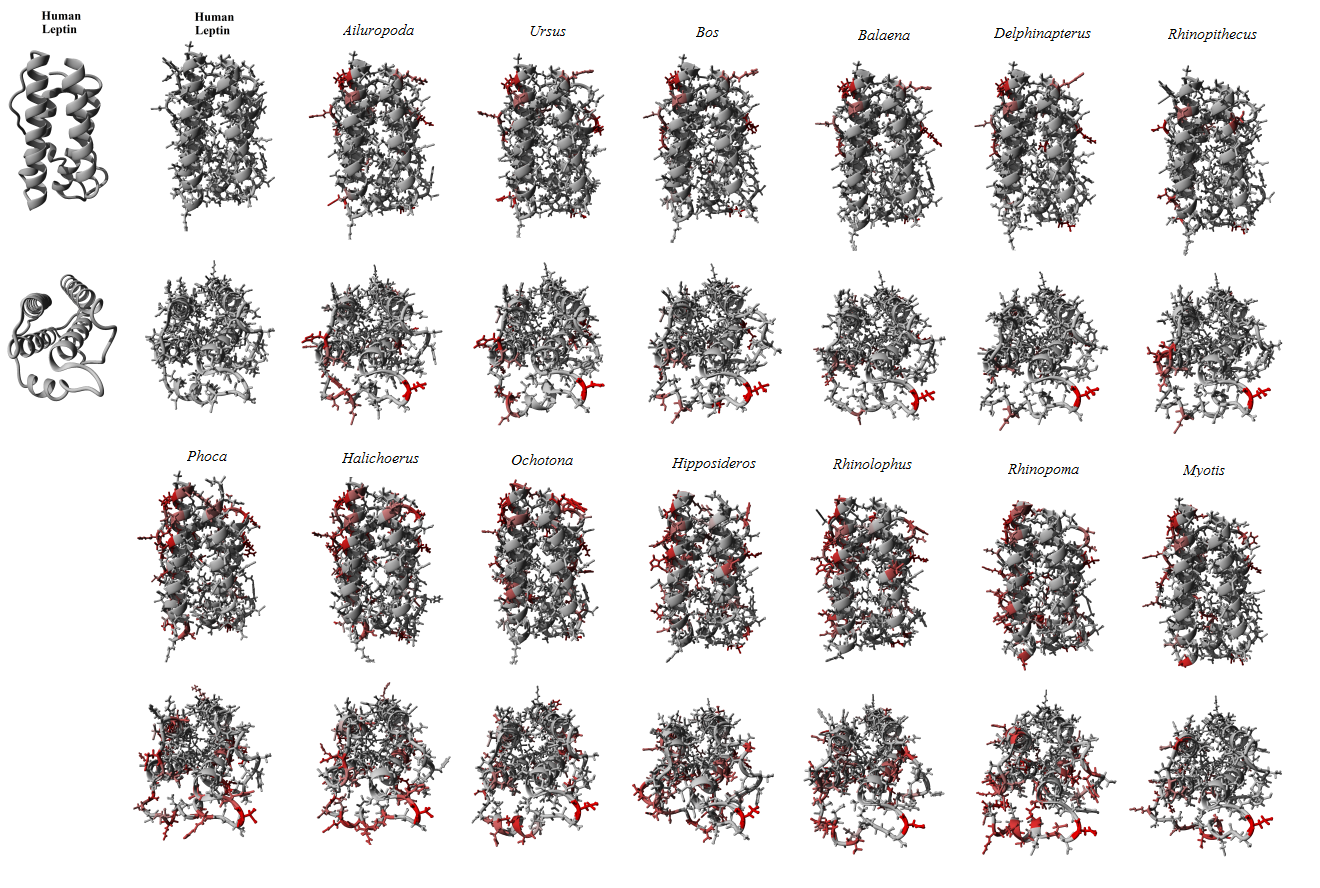

Supplement: Figure S3 — Energy minimized models for each of the 16 sequences. Models showing the amino acids that differ between human and each of the species in red. (TIF) [file pone.0092751.s004.tif]

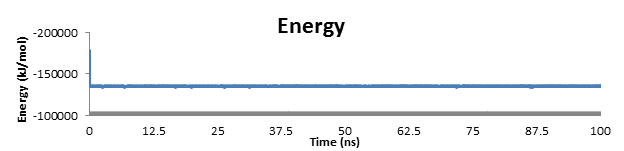

Supplement: Figure S4 — Extended simulation of Peregrine falcon leptin. The energy over the entire simulation showing rapid stabilization. (TIF) [file pone.0092751.s005.tif]

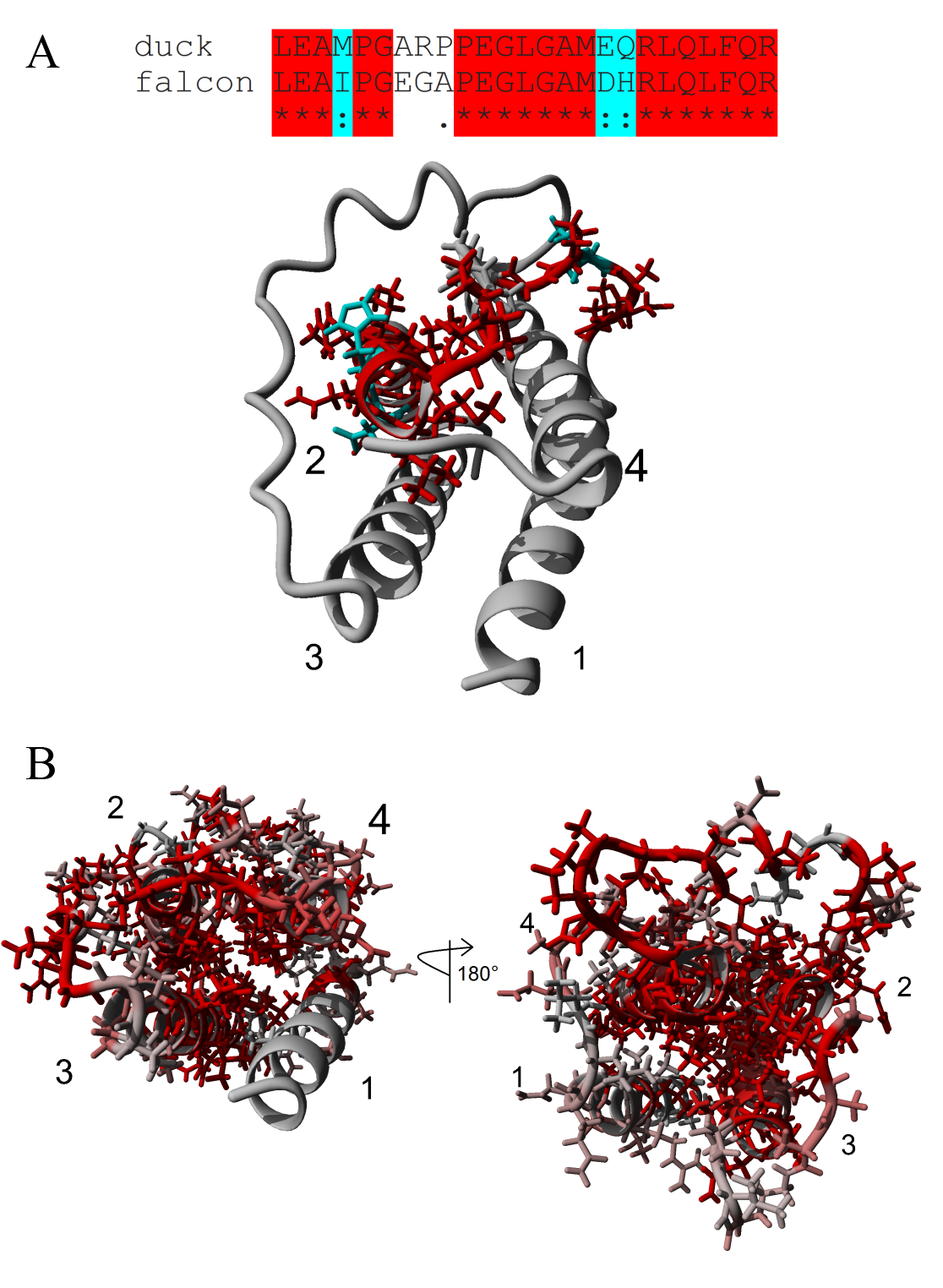

Supplement: Figure S5 — Mapping conservation of avian-specific sequence to the structure of Peregrine falcon. A) Short sequence translated from the duck leptin transcript aligned to falcon. Amino acids shown correspond to the duck transcript and amino acid colors are of that in the sequence alignment. The amino acids correspond to the second helix and the segment that crosses the first helix to the second helix. B) Conserved amino acids (red) between the Peregrine falcon and zebra finch sequences. Sequences for the zebra finch starts at the end of helix one. (TIF) [file pone.0092751.s006.tif]

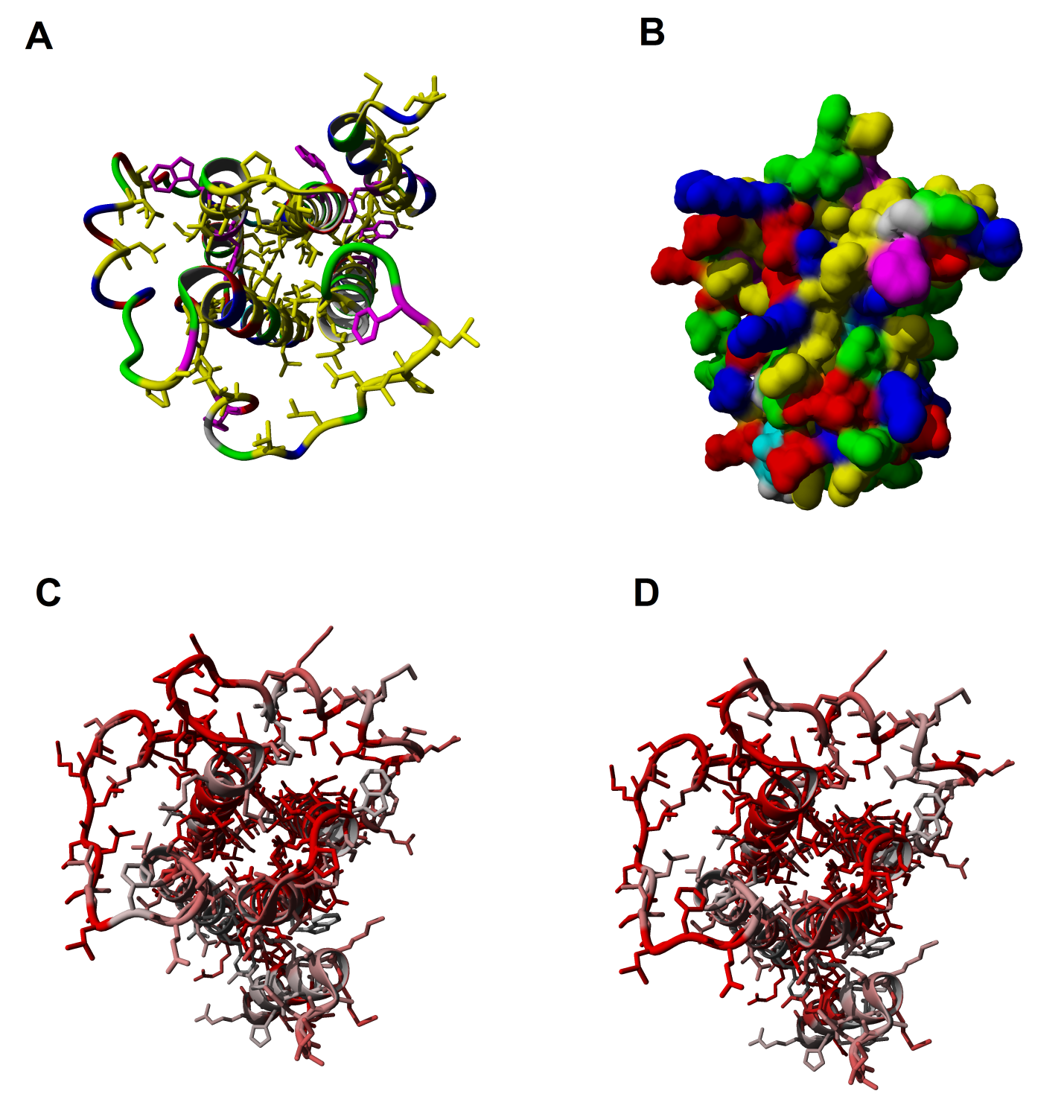

Supplement: Figure S6 — Coelacanth leptin. A) Leptin model with amino acid properties colored (yellow = hydrophobic, magenta = aromatic, red = polar acidic, blue = polar basic, green = hydrophilic) looking down the hydrophobic core. B) Surface plot showing the electrostatic region for interaction with receptor. C) Conserved amino acids (red) between coelacanth and Chelonia sequence shown on the structure of coelacanth. D) Conserved amino acids (red) between coelacanth and Xenopus sequence shown on the structure of coelacanth. (TIF) [file pone.0092751.s007.tif]

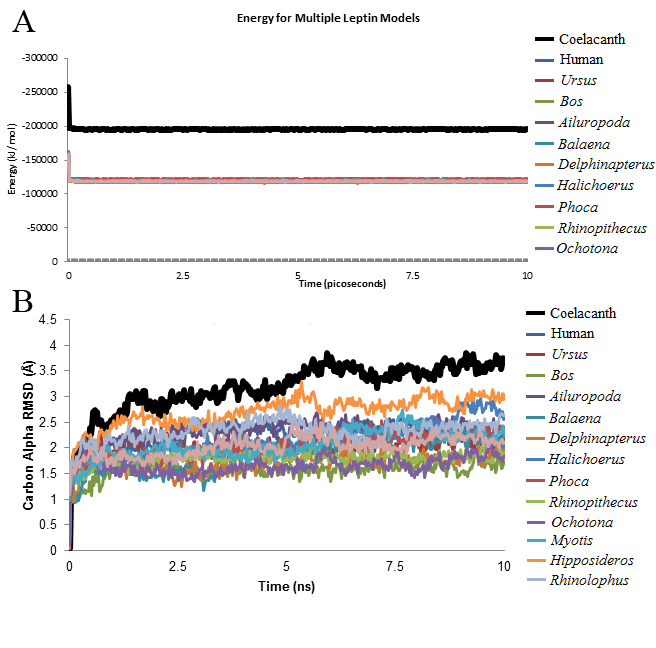

Supplement: Figure S7 — Molecular dynamic simulation for Coelacanth leptin. A) Energy for the Coelacanth over the 10 ns simulation. Energy is very different than the other 15 models used in analysis. B) Molecular dynamic simulations for coelacanth yielded carbon alpha RMSD higher than other species. Both the energy and the movement are consistent with a more open protein structure, typical of cold-bodied animals [43]. (TIF) [file pone.0092751.s008.tif]

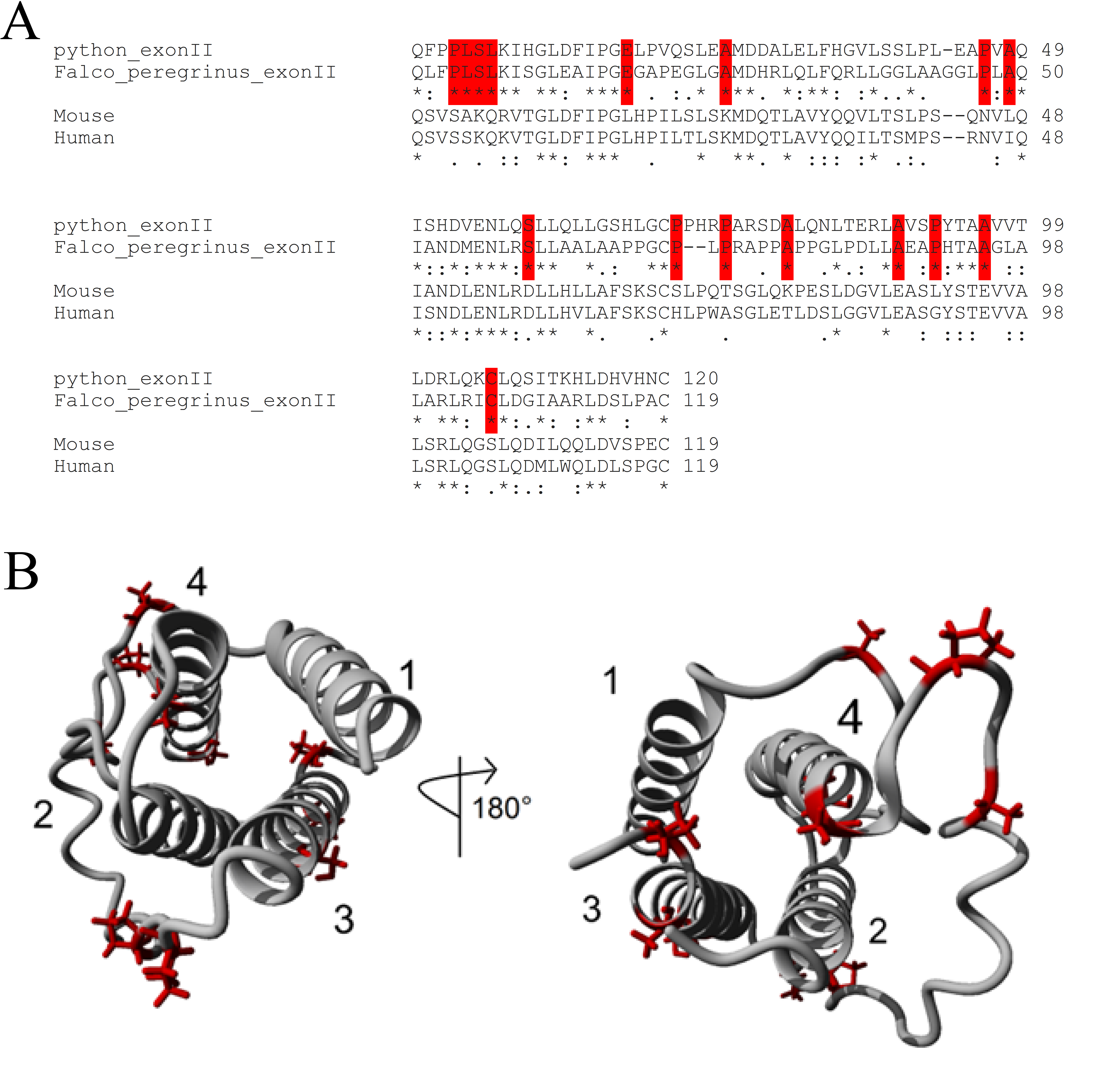

Supplement: Figure S8 — Python leptin. A) Sequence alignment of the python leptin to the Peregrine falcon, mouse and human. Amino acids conserved in python and Peregrine falcon but not the mouse and the human are highlighted in red. B) Amino acids identified in red in A shown on the structure of the Peregrine falcon leptin. These amino acids do not correspond to structural packing. (TIF) [file pone.0092751.s009.tif]

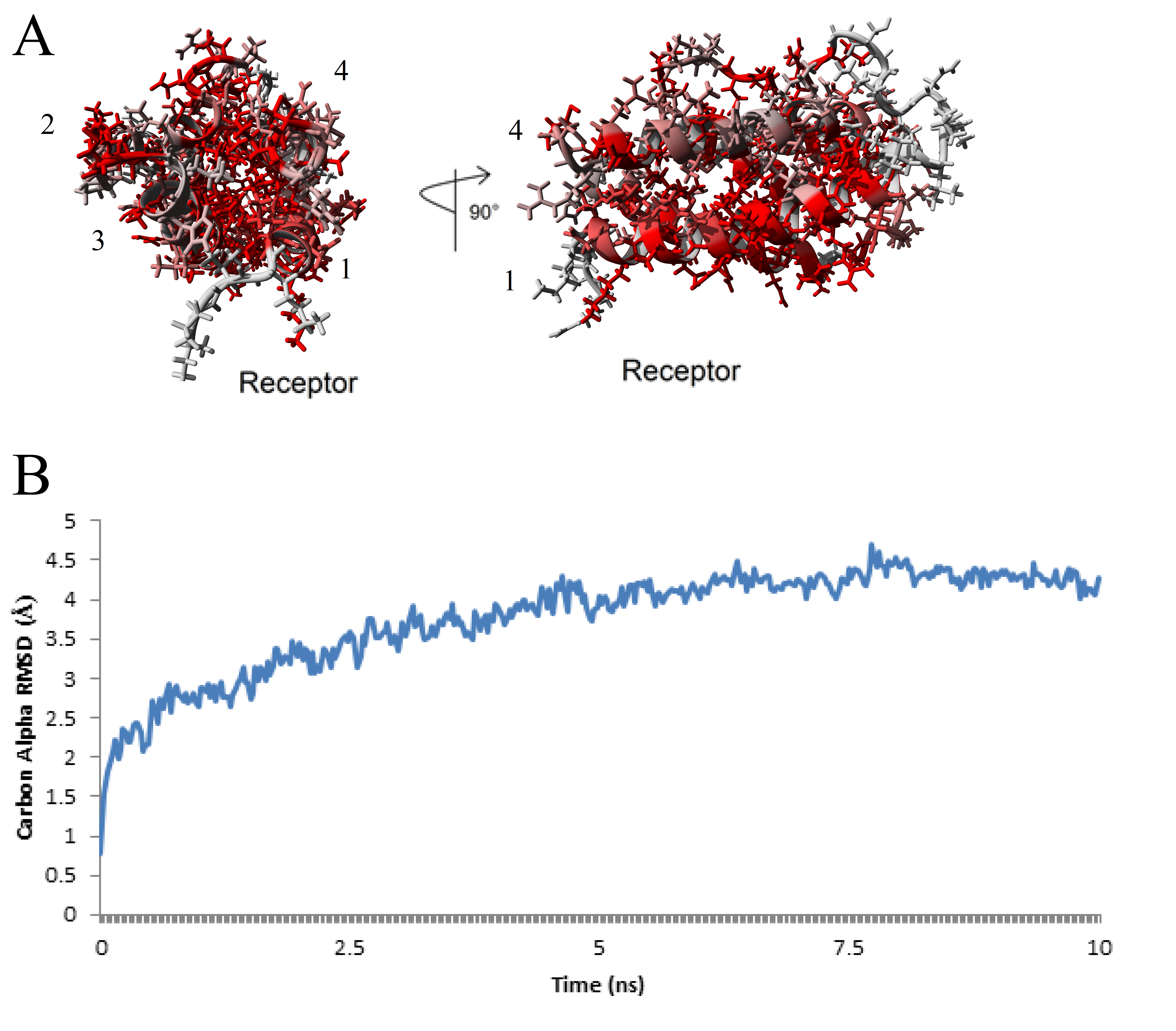

Supplement: Figure S9 — Alligator leptin model. A) Energy minimized model of alligator leptin showing the amino acids that are conserved with Peregrine falcon leptin shown in red. The binding site with the receptor is highly conserved between the two species. B) 10 ns of simulation show stability of the protein structure. (TIF) [file pone.0092751.s010.tif]

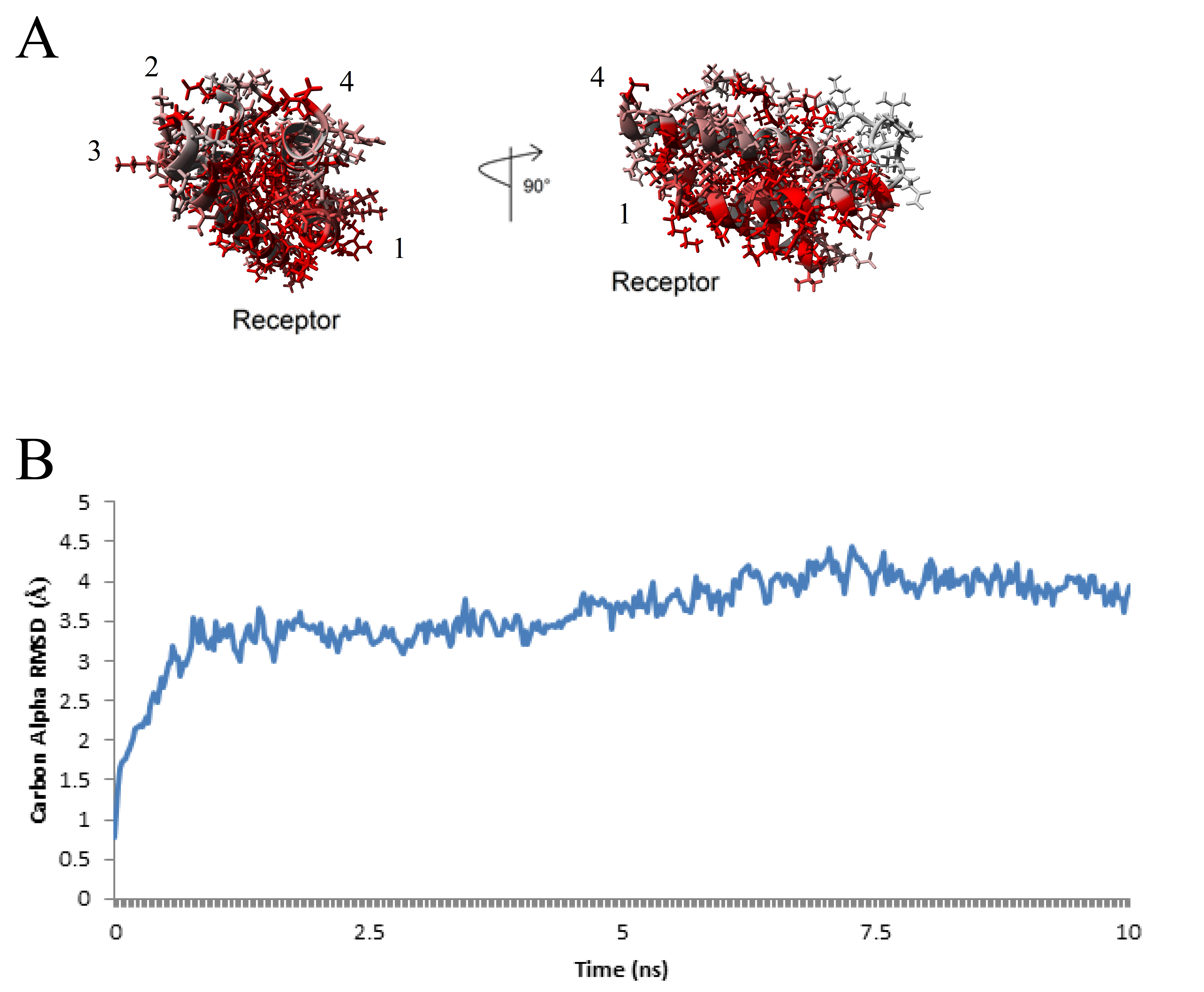

Supplement: Figure S10 — Chelonia leptin model. A) Energy minimized model of Chelonia leptin showing the amino acids that are conserved with Peregrine falcon leptin shown in red. The binding site with the receptor is highly conserved between the two species. B) 10 ns of simulation show stability of the protein structure. (TIF) [file pone.0092751.s011.tif]

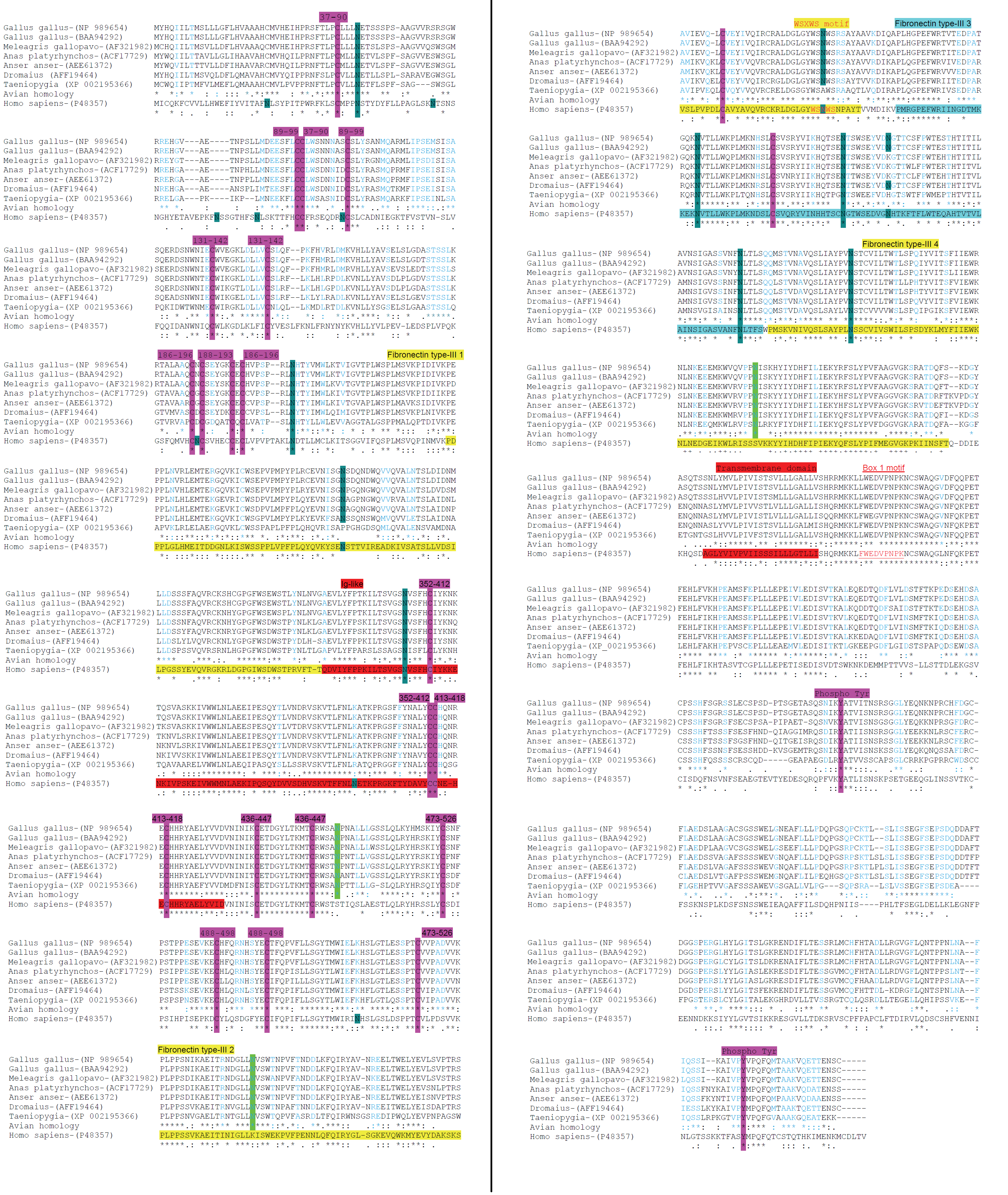

Supplement: Figure S11 — Sequence alignments of avian species with human for leptin receptor. Amino acids colored cyan are conserved in the multiple avian sequences but not with human. Amino acids highlighted in magenta are cysteines known to form disulfide bonds. The WSXWS motif in underlined and colored red. Domains are identified by highlighting the human sequence in yellow, red or cyan. Amino acids known to be N-glycosylated are highlighted in green and those suggested to be N-glycosylated in avian sequences in light green. (TIF) [file pone.0092751.s012.tif]

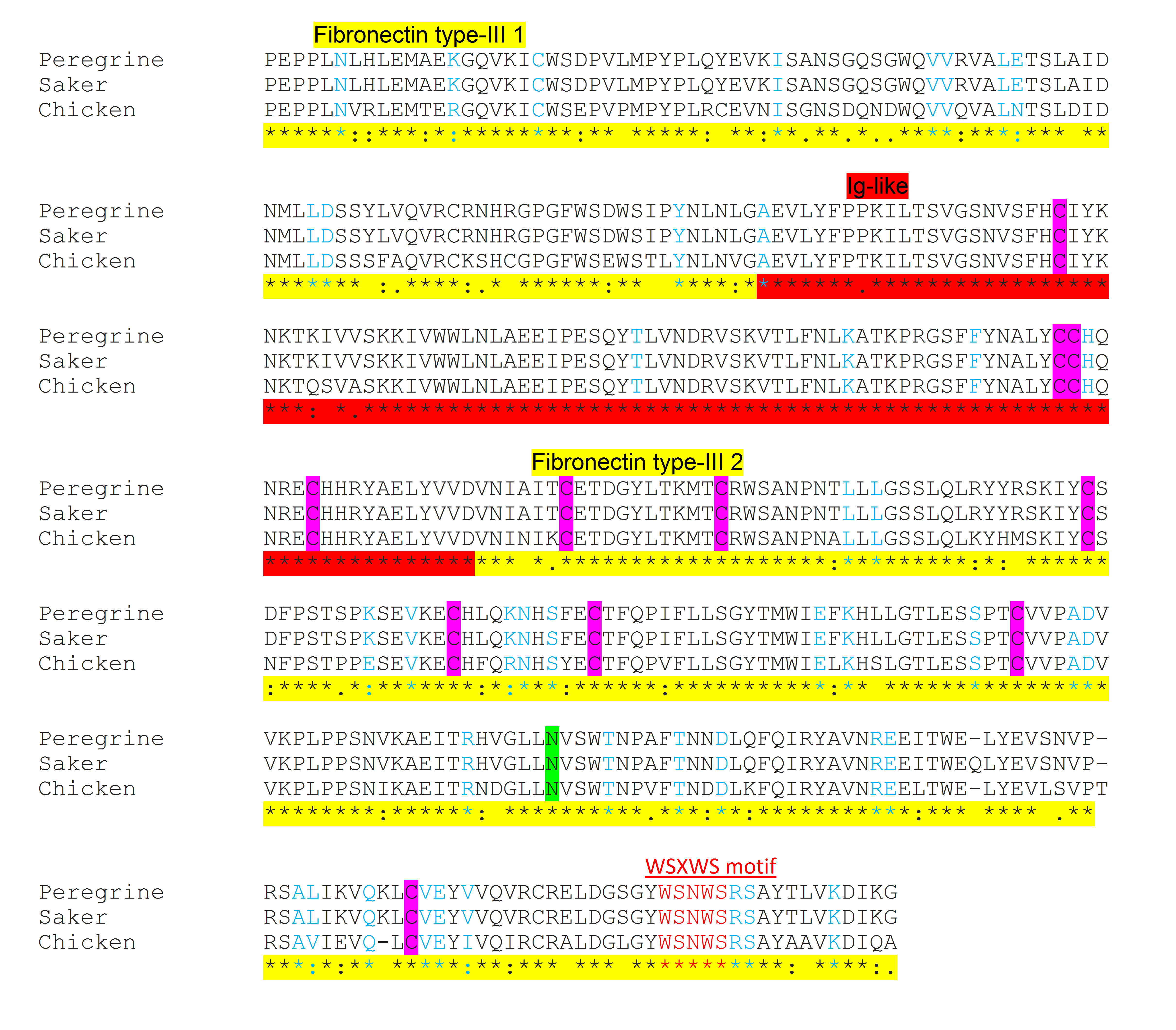

Supplement: Figure S12 — Sequences of the Peregrine and Saker falcon leptin receptor aligned to chicken. Color coding and alignments are the same as in Figure S11. (TIF) [file pone.0092751.s013.tif]

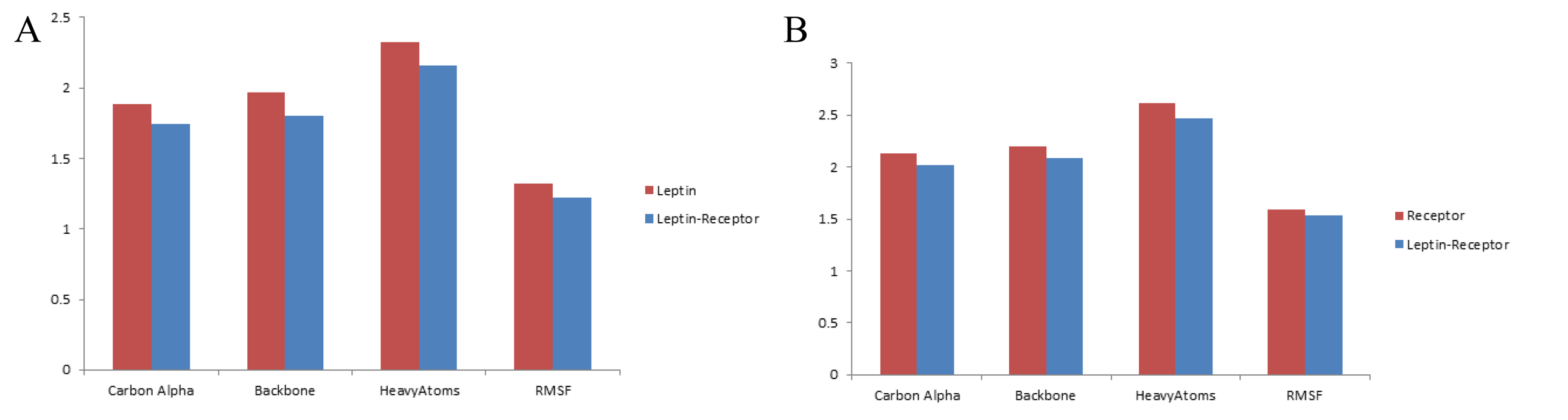

Supplement: Figure S13 — Simulation data for the Peregrine falcon leptin and leptin receptor. A) RMSD following 10 ns simulation for leptin free (red) or complexed to the receptor (blue). B) RMSD following 10 ns simulation for leptin receptor free (red) or complexed to the leptin (blue). Both simulations were stabilized when found as a complex. (TIF) [file pone.0092751.s014.tif]

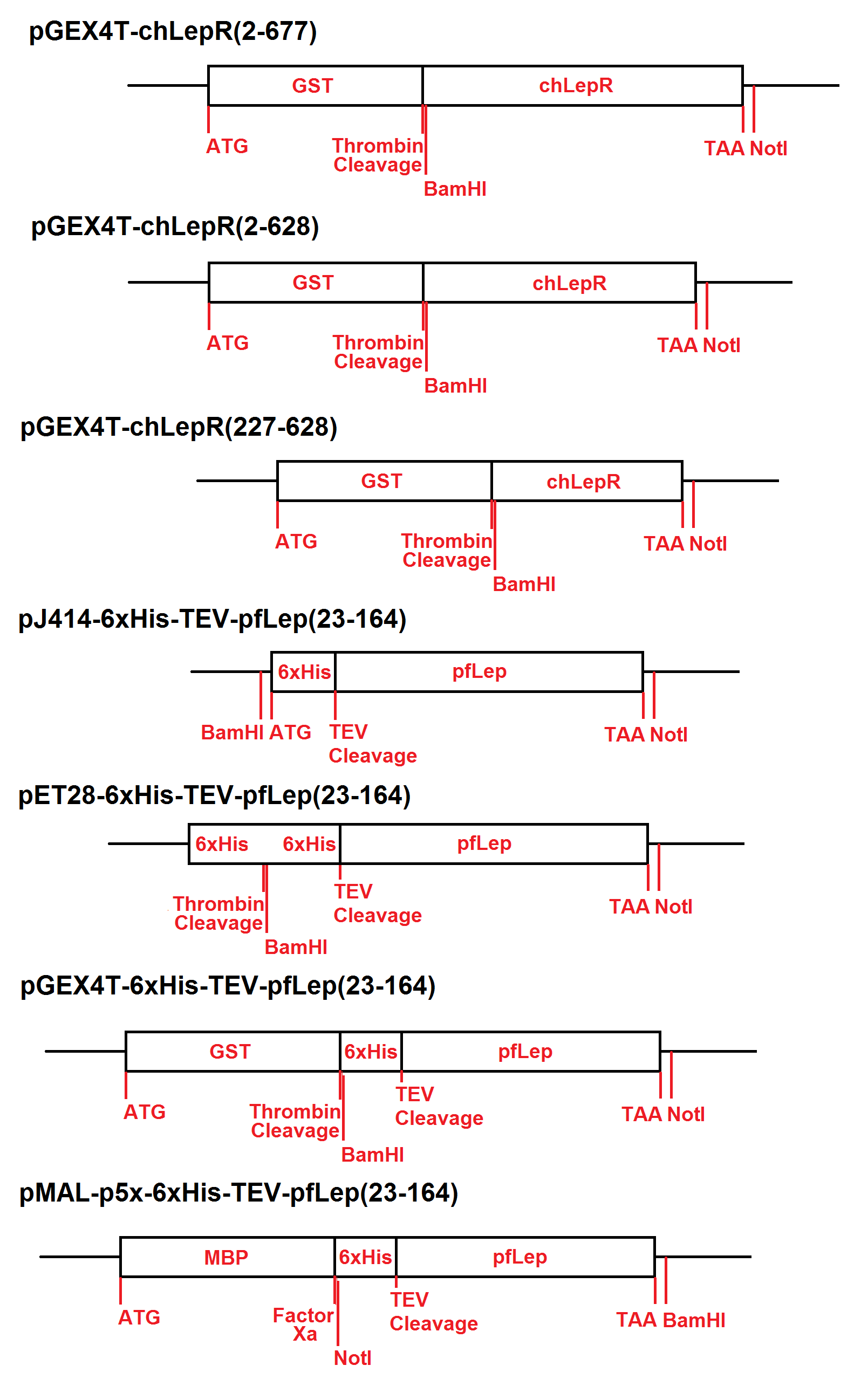

Supplement: Figure S14 — Constructs used for studying avian leptin. Restriction enzymes listed (NotI and BamHI) were used to clone each construct at positions shown on each vector. All constructs contain a cleavable tag when treated with various enzymes (Thrombin, TEV, and Factor Xa). (TIF) [file pone.0092751.s015.tif]
